# Phenolic Composition and Antioxidant Properties of Different Peach [*Prunus persica* (L.) Batsch] Cultivars in China

**DOI:** 10.3390/ijms16035762

**Published:** 2015-03-12

**Authors:** Xiaoyong Zhao, Wenna Zhang, Xueren Yin, Mingshen Su, Chongde Sun, Xian Li, Kunsong Chen

**Affiliations:** 1 Laboratory of Fruit Quality Biology, Zhejiang University, Zijingang Campus, Hangzhou 310058, Zhejiang, China; E-Mails: zhaoxiaoyong-1989@163.com (X.Z.); nawen2007@163.com (W.Z.); xuerenyin@zju.edu.cn (X.Y.); adesun2006@zju.edu.cn (C.S.); akun@zju.edu.cn (K.C.); 2 The State Agriculture Ministry Laboratory of Horticultural Plant Growth, Development and Quality Improvement, Zhejiang University, Zijingang Campus, Hangzhou 310058, Zhejiang, China; 3 Zhejiang Key Laboratory for Agro-Food Processing, Zhejiang University, Zijingang Campus, Hangzhou 310058, Zhejiang, China; 4 Forestry and Pomology Research Institute, Shanghai Academy of Agricultural Sciences, Shanghai 20l403, China; E-Mail: mingshensu@hotmail.com

**Keywords:** *Prunus persica*, phenolic compounds, antioxidant capacities, taxonomy

## Abstract

China is an important centre of diversity for *Prunus persica*. In the present study, 17 Chinese peach cultivars were evaluated for phenolic content and antioxidant activity. Neochlorogenic acid (NCHA), chlorogenic acid (CHA), procyanidin B1 (B1), catechin (CAT), cyanidin-3-*O*-glucoside (C3G), quercetin-3-*O*-galactoside (Q3GAL), quercetin-3-*O*-glucoside (Q3GLU), quercetin-3-*O*-rutinoside (Q3R), and kaempferol-3-*O*-rutinoside (K3R) were identified and quantified. CHA and CAT were the predominant components in both the peel and pulp of this fruit. In general, peel extracts showed higher antioxidant activities than the pulp counterparts, consistent with the observed higher phenolic content. The melting peach cultivar “Xinyu” showed the highest antioxidant potency composite (APC) index. The principal component analysis (PCA) of peel phenolics showed a clear distinction between the melting peach and nectarine. Overall, peach cultivars rich in hydroxycinnamates and flavan-3-ols showed relatively higher antioxidant activities and might be excellent sources of phytochemicals and natural antioxidants.

## 1. Introduction

Epidemiological studies have shown that the consumption of fruit and vegetables has health benefits against chronic diseases, such as cardiovascular disease, cancer, and diabetes [1,2,3]. The health-promoting properties of fruits and vegetables are mainly due to the presence of different antioxidant components, including phenolics [4,5].

Phenolic compounds are a large group of plant secondary metabolites. So far, more than 8000 dietary phenolics have been identified, and their distribution and accumulation profiles can be affected by both genetic and environmental factors [2,4]. Interestingly, distinctive phenolic profiles can be used as taxonomic markers [6,7].

Peaches (*Prunus persica* (L.) Batsch) are nutritionally and economically important and they are one of the most popular fruits consumed worldwide. Peach originated from China more than 4000 years ago and there are more than 3000 peach cultivars in the world today, which can be variously classified as melting and non-melting flesh, or hairy and smooth skin, or clingstone and freestone, *etc.* [8]. Such a huge range of cultivars provides important genetic resources for the evaluation of the phenolic profile. So far, phenolic compounds have been characterized in peach germplasms grown in different regions, such as USA [9,10,11], Italy [12], Spain [13,14], Brazil [15] and Pakistan [16]. As a result, various phenolic compounds have been identified in peach fruits [17,18].

As the largest producer of peach fruits in the world, China (11.9 million metric tons, 2013 FAO data) currently has approximately more than 1000 peach cultivars [19]. However, no extensive investigation of the phenolic profile and nutritional value of Chinese peach cultivars has yet been carried out.

In southern China, melting peaches are famous for their soft texture, juicy flesh, good flavour and sweet taste, which makes them quite competitive in the fresh fruit market. The objective of the present study was to characterize the phenolic compounds in 17 peach cultivars, including 13 melting peach cultivars grown in southern China, and to evaluate their antioxidant capacities. Such results may help to select new genotypes rich in phenolic content and enhanced nutritional properties and to provide useful information for the utilization of peach genetic resources.

## 2. Results and Discussion

### 2.1. Fruit Quality Evaluation

All fruits used in the present study were harvested at the ready-to-eat stage. As shown in Table 1, fruit quality indices, such as fresh weight (FW), fruit shape index (FSI), and soluble solids content (SSC), varied significantly among the 17 cultivars tested. The melting peach cultivar SZZS showed the highest FW value (233.94 g), while the nectarine cultivar HY002 showed the lowest value (83.61 g). The FSI values varied from 0.89 (QSBT) to 1.14 (HY002). SSC is an important fruit quality trait, which is closely related to consumer acceptance and satisfaction. In this study, the SSC of the 17 peach cultivars ranged from 8.34 (ANSM) to 15.48 °Brix (YL). Low SSC values were observed for several early maturing cultivars such as ANSM, HY004, and ZX, and similar observation was also found in early maturing cultivars such as UFO-2, UFO-3, and UFO-4 grown in Spain [20]. Different peach cultivars grown in California [11] and Italy [21] also showed significant variations in fruit quality indices, such as SSC and titratable acid.

**Table 1:** Cultivars used in the study and their respective quality indices.

Number	Cultivars	Abbreviation	Fruit Type	Flesh Colour	FW (g)	FSI	SSC (°Brix)
1	Annongshuimi	ANSM	Melting	White	179.55 ± 18.55 ^c^	0.97 ± 0.05 ^d^	8.34 ± 1.06 ^f^
2	Chunfeng	CF	Melting	White	122.59 ± 12.30 ^g^	1.12 ± 0.06 ^a^	8.75 ± 0.85 ^e,f^
3	Chiyue	CY	Melting	White	181.47 ± 8.56 ^c^	0.97 ± 0.03 ^d^	11.27 ± 1.34 ^c^^,d^
4	Danxia	DX	Melting	White	181.85 ± 10.92 ^c^	0.96 ± 0.04 ^d^	10.92 ± 0.72 ^c^^,d^
5	Dayubaifeng	DYBF	Melting	White	166.63 ± 11.79 ^d,e^	0.96 ± 0.03 ^d^	11.75 ± 0.53 ^c^
6	Hujingmilu	HJML	Melting	White	200.47 ± 11.95 ^b^	0.95 ± 0.05 ^d^	13.92 ± 1.98 ^b^
7	Jinhuadabaitao	JHDBT	Melting	White	201.32 ± 26.57 ^b^	1.02 ± 0.04 ^c^	8.81 ± 0.92 ^e,f^
8	Qingshuibaitao	QSBT	Melting	White	201.55 ± 10.58 ^b^	0.89 ± 0.02 ^e^	15.30 ± 2.05 ^a^
9	Shazizaosheng	SZZS	Melting	White	233.94 ± 24.01 ^a^	0.98 ± 0.04 ^d^	10.61 ± 1.03 ^d^
10	Wujingzaobaifeng	WJZBF	Melting	Red	136.66 ± 7.70 ^f^	0.95 ± 0.03 ^d^	9.30 ± 1.23 ^e,f^
11	Xinyu	XY	Melting	White	209.98 ± 13.64 ^b^	1.02 ± 0.03 ^c^	14.10 ± 0.73 ^b^
12	Yulu	YL	Melting	White	198.84 ± 17.24 ^b^	0.95 ± 0.04 ^d^	15.48 ± 1.03 ^a^
13	Zhaoxia	ZX	Melting	White	172.05 ± 18.55 ^c^^,d^	0.90 ± 0.05 ^e^	8.45 ± 1.94 ^f^
14	Huyou002	HY002	Nectarine	White	83.61 ± 6.22 ^i^	1.14 ± 0.04 ^a^	9.45 ± 0.69 ^e,f^
15	Huyou003	HY003	Nectarine	Yellow	100.57 ± 11.39 ^h^	0.97 ± 0.03 ^d^	9.57 ± 0.78 ^e^
16	Huyou004	HY004	Nectarine	Yellow	135.85 ± 8.07 ^f^	1.02 ± 0.04 ^c^	8.42 ± 1.06 ^f^
17	Huyou018	HY018	Nectarine	Yellow	157.83 ± 13.62 ^e^	1.06 ± 0.05 ^b^	9.21 ± 1.12 ^e,f^

Abbreviations: FW, fresh weight; FSI, fruit shape index; SSC, soluble solid content; Data were expressed as the means ± standard deviation of twelve samples; Different superscripts in the same column represent significant differences (*p* < 0.05).

### 2.2. Identification of Individual Phenolic Compound

Identification of individual phenolic compounds in peach fruit was carried out by HPLC-DAD and LC-ESI-MS/MS. For the identification of hydroxycinnamates, the fragment ion information from LC-MS/MS was compared with a previous study [22]. As a result, two hydroxycinnamates were identified in peach fruit (Table 2). They both showed the same [M − H]^−^ ion at *m*/*z* 353.1, the [quinic − H]^−^ ion at *m*/*z* 191.1, and the [caffic − H]^−^ ion at *m*/*z* 179.0, indicating that these two compounds were isomers with the same molecular weight of 354. Further linkage position of caffic residues on the quinic acid were analyzed according to the rules reported by Clifford *et al.* [22], and together with the confirmation of chemical standards, they were identified as neochlorogenic acid (NCHA) and chlorogenic acid (CHA), respectively.

**Table 2:** Identification of phenolic compounds in peach fruits in negative ions with HPLC-DAD and LC-ESI-MS/MS.

Phenolic Groups	λ_max_ (nm)	Molecular Weight	MS^2^ (*m*/*z*)	Tentative Identification	R Groups
Hydroxycinnamates 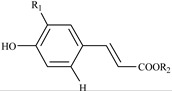	324.6, 240.4	354	353.1, 191.1, 179.0	NCHA	R_1_ = OH; R_2_ = 5-quinic acid
	327.0, 241.6	354	353.1, 191.1, 179.0	CHA	R_1_ = OH; R_2_ = 3-quinic acid
Flavan-3-ols 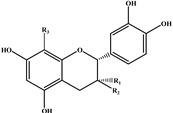	279.4	578	577.1, 425.1, 407.1, 289.1	B1	R_1_ = H; R_2_ = OH; R_3_ = epicatechin
	279.4	290	289.0	CAT	R_1_ = H; R_2_ = OH; R_3_ = H
Anthocyanins 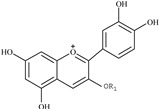	514.9, 279.4	449	447.0, 284.9	C3G	R_1_ = glucoside
Flavonols 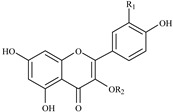	355.7, 254.6	464	463.1, 301.0, 300.0	Q3GAL	R_1_ = OH; R_2_ = galactoside
	356.9, 255.7	464	463.1, 301.0, 300.0	Q3GLU	R_1_ = OH; R_2_ = glucoside
	355.7, 255.7	610	609.2, 301.0, 300.0	Q3R	R_1_ = OH; R_2_ = rutinoside
	347.3, 265.2	594	593.1, 285.0	K3R	R_1_ = H; R_2_ = rutinoside

For the identification of flavan-3-ols, the fragment ion information from LC-MS/MS was compared with the study of Sanz *et al.* [23]. As a consequence, two flavan-3-ols were identified in peach fruit (Table 2). The [M − H]^−^ ion at *m*/*z* 289.0 indicated a structure of monomeric flavanol. The other one showed [M − H]^−^ ion at *m*/*z* 577.1, suggesting the molecular weight of a procyanidin dimer with a B-type interflavanoid linkage. As expected for procyanidins, retro Diels-Alder (RDA) fission of the heterocyclic rings of dimeric procyanidins occurred and resulted in the fragment *m*/*z* 425.1. The product of the subsequent water elimination (*m*/*z* 407.1) was also detected in significant amounts. The cleavage of the interflavonoid linkage leading to *m*/*z* 289.1 [M − H − 288]^−^ was also observed. Further analyses were conducted according to the rules reported by Sanz *et al.* [23], and together with the confirmation of chemical standards and their typical UV profiles, they were identified as catechin (CAT) and procyanidin B1 (B1), respectively.

For the identification of anthocyanins, the fragment ion information from LC-MS/MS was compared with the study of Tomás-Barberán *et al.* [18]. As a result, one anthocyanin was identified in peach fruit (Table 2). It showed [M − H]^−^ ion at *m*/*z* 447.0, which indicated a molecular weight of 449, and the cleavage of the interflavonoid linkage leading to *m*/*z* 284.9 [M − H − 162]^−^ was also observed. This compound was identified as cyanidin-3-*O*-glucoside (C3G) and was confirmed with its chemical standard.

For the identification of flavonols, the fragment ion information from LC-MS/MS was compared with a previous study [24]. Consequently, four flavonols were identified (Table 2). Among them, three quercetin glucosides showed [M − H]^−^ ions at *m*/*z* 463.1 or 609.2, which indicated a molecular weight of 464 or 610, and they all showed a [quercetin − H]^−^ ion at *m*/*z* 301.0 and a [quercetin − 2H]^−^ ion at *m*/*z* 300.0. One kaempferol glucoside showed [M − H]^−^ ion at *m*/*z* 593.1 indicating the molecular weight of 594, and it showed a [kaempferol − H]^−^ ion at *m*/*z* 285.0. Further glucosides type and linkage position of glycosides on the quercetin or kaempferol were analyzed according to the rule reported by Hvattum and Ekeberg [24], and together with the confirmation of chemical standards, four flavonols were identified as quercetin-3-*O*-galactoside (Q3GAL), quercetin-3-*O*-glucoside (Q3GLU), quercetin-3-*O*-rutinoside (Q3R), and kaempferol-3-*O*-rutinoside (K3R), respectively.

### 2.3. Quantification of Phenolic Compounds

Due to the significant correlations observed between the phenolic contents and various bioactivities, numerous studies have been conducted to select new genotypes rich in phenolic compounds and enhanced nutritional properties [9,13,14].

In the present study, nine phenolic compounds, including two hydroxycinnamates (NCHA and CHA), two flavan-3-ols (B1 and CAT), one anthocyanin (C3G) and four flavonols (Q3GAL, Q3GLU, Q3R, K3R), were quantified in the peel and pulp of the 17 peach cultivars (Table 3 and Table 4). In general, the peel extracts contained higher amounts of phenolics than the pulp counterparts, and anthocyanin and flavonols primarily accumulated in the peel. CHA and CAT were present at much higher concentrations than other phenolic compounds in both parts of the fruit (Table 3 and Table 4).

NCHA and CHA were the primary hydroxycinnamates detected in Chinese peach fruit, and CHA was predominantly observed. The CHA contents varied from 52.20 (ANSM) to 1631.25 mg/kg DW (HY002) in the peel and from 27.50 (ZX) to 568.07 mg/kg DW (CY) in the pulp. The NCHA contents varied from 5.77 (ANSM) to 342.75 (XY) mg/kg DW in the peel and from 15.74 (ZX) to 267.78 (XY) mg/kg DW in the pulp. In the peel, HY002, HY018, DX, QSBT, CY, DYBF, HY004 and XY were rich in CHA (>1000 mg/kg DW). In the pulp, the amount of CHA in CY was nearly 20 times higher than that in ZX. The presence of CHA as the primary phenolic compound has also been observed in previous studies [15,18].

B1 and CAT were the major flavan-3-ol compounds detected in the Chinese peach cultivars and CAT was the dominant one. The B1 content varied from undetectable to 539.22 mg/kg DW (XY) in the peel and 403.75 mg/kg DW (XY) in the pulp. The CAT contents ranged from 60.14 (ANSM) to 1030.06 mg/kg DW (HY018) in the peel and from undetectable to 374.43 mg/kg DW (CY) in the pulp, indicating a large variation among different cultivars.

**Table 3:** Contents of nine phenolic compounds (mg/kg DW) in the peel of 17 *P. persica* cultivars.

Cultivars	Hydroxycinnamates		Flavan-3-ols		Anthocyanin	Flavonols			
	NCHA	CHA	B1	CAT	C3G	Q3GAL	Q3GLU	Q3R	K3R
ANSM	5.77 ± 0.51 ^j^	52.20 ± 0.76 ^k^	nd	60.14 ± 8.39 ^j^	9.33 ± 0.73 ^g^	15.52 ± 1.19 ^j^	16.37 ± 1.53 ^h–j^	120.16 ± 6.09 ^e,f^	51.17 ± 4.08 ^c^
CF	138.72 ± 5.89 ^g^	609.27 ± 25.74 ^h^	54.76 ± 2.10 ^h^	474.88 ± 29.90 ^f^^,g^	134.66 ± 11.72 ^e^	62.08 ± 2.56 ^d,e^	58.64 ± 3.04 ^e,f^	193.25 ± 5.94 ^a^	110.86 ± 4.43 ^a^
CY	193.02 ± 3.31 ^d^	1090.86 ± 19.57 ^d,e^	496.26 ± 16.13 ^a^^,b^	768.15 ± 45.85 ^c^^,d^	25.39 ± 1.91 ^g^	24.09 ± 4.71 ^h–j^	15.04 ± 0.43 ^i,j^	78.32 ± 0.79 ^j^	29.45 ± 0.60 ^g^^,h^
DX	219.13 ± 4.57 ^c^	1166.15 ± 15.73 ^c^	295.16 ± 33.03 ^e^	514.75 ± 38.27 ^e,f^	69.27 ± 7.76 ^f^	17.86 ± 0.26 ^i,j^	55.44 ± 11.94 ^e,f^	69.82 ± 1.74 ^k^	39.51 ± 0.36 ^e^
DYBF	195.74 ± 5.90 ^d^	1051.63 ± 23.82 ^d,e^	324.11 ± 9.70 ^d,e^	576.17 ± 15.80 ^e^	19.15 ± 2.32 ^g^	42.02 ± 1.12 ^f^^,g^	27.15 ± 0.41 ^h,i^	85.67 ± 0.26 ^i^	29.79 ± 0.49 ^g^^,h^
HJML	130.05 ± 1.57 ^g^	720.93 ± 10.47 ^g^	206.17 ± 36.62 ^f^	428.95 ± 18.97 ^g^	24.75 ± 1.63 ^g^	22.98 ± 0.47 ^h–j^	20.19 ± 1.28 ^h–j^	74.24 ± 1.17 ^j,k^	32.50 ± 0.53 ^f^^–h^
JHDBT	32.04 ± 1.61 ^i^	162.72 ± 8.64 ^j^	nd	149.69 ± 25.47 ^i^	11.65 ± 0.49 ^g^	23.98 ± 1.13 ^h–j^	23.08 ± 1.00 ^h–j^	115.82 ± 2.84 ^f^^,g^	33.02 ± 1.27 ^f^^,g^
QSBT	171.16 ± 6.74 ^e^	1110.77 ± 46.02 ^c^^,d^	452.63 ± 39.33 ^b^	806.87 ± 36.87 ^c^	nd	33.53 ± 1.04 ^g^^–i^	31.67 ± 1.14 ^g^^–i^	126.09 ± 7.63 ^d,e^	34.83 ± 0.86 ^f^
SZZS	87.61 ± 2.98 ^h^	329.71 ± 6.93 ^i^	nd	253.46 ± 18.26 ^h^	21.00 ± 0.58 ^g^	52.66 ± 2.36 ^e,f^	52.29 ± 2.34 ^e–g^	139.54 ± 3.51 ^c^	39.62 ± 1.60 ^e^
WJZBF	136.15 ± 4.68 ^g^	674.38 ± 25.83 ^g^^,h^	120.65 ± 16.94 ^g^	483.52 ± 56.84 ^f^^,g^	220.30 ± 11.40 ^d^	69.38 ± 2.77 ^d^	67.84 ± 2.86 ^e^	172.73 ± 6.07 ^b^	44.87 ± 1.63 ^d^
XY	342.75 ± 16.06 ^a^	1020.50 ± 51.80 ^e,f^	539.22 ± 45.95 ^a^	707.32 ± 82.98 ^d^	18.57 ± 1.59 ^g^	20.26 ± 3.89 ^i,j^	14.00 ± 2.63 ^i,j^	75.72 ± 4.30 ^j,k^	34.54 ± 4.43 ^f^
YL	166.62 ± 8.99 ^e,f^	955.83 ± 49.43 ^f^	368.75 ± 47.92 ^c^^,d^	573.79 ± 25.82 ^e^	nd	8.45 ± 0.57 ^j^	2.45 ± 0.21 ^j^	59.15 ± 0.65 ^l^	28.81 ± 0.73 ^h^
ZX	10.56 ± 0.43 ^j^	126.72 ± 6.50 ^j^	nd	196.62 ± 18.36 ^h,i^	28.55 ± 5.12 ^g^	36.61 ± 1.58 ^g^^,h^	39.29 ± 0.86 ^f^^–h^	90.80 ± 1.37 ^i^	55.64 ± 1.18 ^b^
HY002	257.16 ± 9.73 ^b^	1631.25 ± 71.17 ^a^	153.71 ± 35.96 ^g^	748.86 ± 40.54 ^c^^,d^	304.78 ± 37.12 ^c^	396.49 ± 19.96 ^a^	581.21 ± 18.09 ^a^	131.76 ± 2.97 ^d^	29.85 ± 0.84 ^g^^,h^
HY003	95.54 ± 1.43 ^h^	741.79 ± 18.35 ^g^	203.44 ± 8.24 ^f^	911.35 ± 21.10 ^b^	125.52 ± 17.51 ^e^	267.38 ± 5.19 ^c^	388.36 ± 8.11 ^d^	111.01 ± 2.18 ^g^^,h^	18.39 ± 0.29 ^i^
HY004	135.63 ± 7.66 ^g^	1037.81 ± 62.36 ^d,e^	108.79 ± 14.09 ^g^	741.83 ± 54.10 ^c^^,d^	398.93 ± 47.39 ^b^	324.67 ± 17.09 ^b^	530.32 ± 26.04 ^c^	109.07 ± 3.17 ^h^	18.64 ± 0.95 ^i^
HY018	157.48 ± 12.15 ^f^	1264.42 ± 99.20 ^b^	384.59 ± 20.18 ^c^	1030.06 ± 39.18 ^a^	670.59 ± 59.63 ^a^	335.07 ± 22.05 ^b^	555.83 ± 37.15 ^b^	106.32 ± 3.54 ^h^	16.91 ± 1.00 ^i^

nd, not detectable; Abbreviations: NCHA, neochlorogenic acid; CHA, chlorogenic acid; B1, procyanidin B1; CAT, catechin; C3G, Cyanidin-3-*O*-glucoside; Q3GAL, quercetin-3-*O*-galactoside; Q3GLU, quercetin-3-*O*-glucoside; Q3R, quercetin-3-*O*-rutinoside; K3R, kaempferol-3-*O*-rutinoside; Data were expressed as the means ± standard deviation of triplicate samples; Different superscripts in the same column represent significant differences (*p* < 0.05).

**Table 4:** Contents of the nine phenolic compounds (mg/kg DW) detected in the pulp tissues of 17 *P. persica* cultivars.

Cultivars	Hydroxycinnamates		Flavan-3-ols		Anthocyanin	Flavonols			
	NCHA	CHA	B1	CAT	C3G	Q3GAL	Q3GLU	Q3R	K3R
ANSM	25.77 ± 1.70 ^j^	41.28 ± 2.50 ^k^	nd	nd	35.44 ± 4.12 ^b^	nd	nd	nd	nd
CF	262.49 ± 13.09 ^a^^,b^	238.78 ± 11.17 ^e,f^	10.28 ± 3.97 ^g^	156.19 ± 11.85 ^e,f^	nd	nd	nd	nd	nd
CY	179.01 ± 6.54 ^d^	568.07 ± 20.91 ^a^	331.64 ± 19.49 ^b^	374.30 ± 11.38 ^a^	nd	nd	nd	nd	nd
DX	174.60 ± 7.09 ^d^	349.04 ± 14.23 ^c^	89.00 ± 8.89 ^e^	123.97 ± 5.18 ^g^	12.86 ± 1.33 ^c^	nd	nd	nd	nd
DYBF	157.44 ± 3.94 ^e^	354.06 ± 9.07 ^c^	142.58 ± 8.82 ^d^	206.65 ± 6.85 ^c^^,d^	nd	nd	nd	nd	nd
HJML	117.20 ± 7.73 ^f^	262.55 ± 15.95 ^e^	62.83 ± 5.18 ^f^	87.71 ± 8.42 ^h^	3.83 ± 0.67 ^d^	nd	nd	nd	nd
JHDBT	57.34 ± 5.02 ^i^	76.99 ± 6.34 ^j^	nd	nd	10.88 ± 1.00 ^c^^,d^	4.90 ± 0.87 ^a^	2.02 ± 0.38 ^b^	nd	nd
QSBT	112.00 ± 11.70 ^f^	291.22 ± 29.12 ^d^	141.27 ± 20.72 ^d^	174.48 ± 15.84 ^d–f^	nd	nd	nd	nd	nd
SZZS	119.13 ± 5.62 ^f^	109.42 ± 3.73 ^i^	nd	nd	18.08 ± 1.03 ^c^	2.93 ± 0.10 ^b^	0.71 ± 0.14 ^b^	nd	nd
WJZBF	110.15 ± 3.80 ^f^	175.89 ± 5.84 ^h^	nd	70.85 ± 1.34 ^h^	184.81 ± 10.14 ^a^	4.90 ± 0.02 ^a^	21.35 ± 1.10 ^a^	nd	nd
XY	267.78 ± 16.47 ^a^	189.14 ± 11.16 ^g^^,h^	403.75 ± 24.5 ^a^	293.32 ± 40.38 ^b^	32.24 ± 5.39 ^b^	nd	nd	nd	nd
YL	251.66 ± 14.99 ^b^	419.35 ± 26.37 ^b^	186.20 ± 9.15 ^c^	190.34 ± 11.65 ^c^^,d^	39.19 ± 2.74 ^b^	nd	nd	nd	nd
ZX	15.74 ± 0.53 ^j^	27.50 ± 0.79 ^k^	nd	nd	nd	nd	nd	nd	nd
HY002	232.36 ± 7.27 ^c^	312.14 ± 15.78 ^d^	nd	nd	nd	nd	nd	nd	nd
HY003	82.86 ± 2.60 ^g^^,h^	129.09 ± 11.28 ^i^	67.89 ± 4.38 ^e,f^	211.16 ± 17.14 ^c^	12.68 ± 0.87 ^c^	nd	nd	nd	nd
HY004	95.92 ± 1.97 ^g^	201.65 ± 13.11 ^g^	13.01 ± 1.69 ^g^	144.36 ± 30.23 ^f^^,g^	4.74 ± 1.10 ^d^	nd	nd	nd	nd
HY018	77.95 ± 5.01 ^h^	232.56 ± 11.76 ^f^	68.26 ± 16.09 ^e,f^	184.60 ± 10.00 ^c^^–e^	38.13 ± 6.27 ^b^	nd	nd	nd	nd

nd, not detectable; Abbreviations: NCHA, neochlorogenic acid; CHA, chlorogenic acid; B1, procyanidin B1; CAT, catechin; C3G, Cyanidin-3-*O*-glucoside; Q3GAL, quercetin-3-*O*-galactoside; Q3GLU, quercetin-3-*O*-glucoside; Q3R, quercetin-3-*O*-rutinoside; K3R, kaempferol-3-*O*-rutinoside; Data were expressed as the means ± standard deviation of triplicate samples; Different superscripts in the same column represent significant differences (*p* < 0.05).

C3G was identified as the main anthocyanin in the peach and is responsible for the red colour in these fruits [18]. A higher C3G content was detected in the peel compared with the pulp. However, small amounts of pigments were also detected in the pulp in some cultivars, particularly in tissues near the stone. In our study, the peel of HY018 (670.59 mg C3G/kg DW) and the pulp of WJZBF (184.81 mg C3G/kg DW C3G) contained the highest amounts of C3G among the peach cultivars examined. Increasing evidence of the benefit of anthocyanins to human nutrition and health has increased research interests in the red flesh colour in peach breeding [9,14].

Q3GAL, Q3GLU, Q3R and K3R were the major flavonols identified in the peach fruit, and Q3GLU was predominantly observed. These flavonols were primarily detected in the peel, and only trace amounts of Q3GAL and Q3GLU were detected in the pulp of some peach cultivars, consistent with previous studies [17]. The peel of four nectarine cultivars showed relatively high Q3GLU contents, ranging from 388.36 (HY003) to 581.21 mg/kg DW (HY002), while the melting peach cultivars showed a relatively low Q3GLU content, with values below 70 mg/kg DW. Similarly, the peels of four nectarine cultivars showed relatively high Q3GAL contents, ranging from 267.38 (HY003) to 396.49 mg/kg DW (HY002), while the melting peach cultivars showed a relatively low Q3GAL content, with values below 70 mg/kg DW. Q3R and K3R were only detected in the peels of peach fruits, ranging in concentration from 59.15 to 193.25 mg/kg DW for Q3R and from 16.91 to 110.86 mg/kg DW for K3R in the cultivars tested.

### 2.4. Variation Patterns and Principal Components Analysis (PCA)

**Figure 1:**
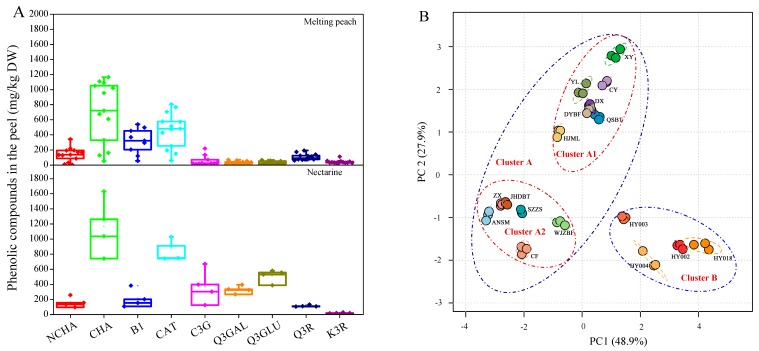
Analysis of the phenolic contents in the peel of 17 *P. persica* cultivars (**A**) and the classification of different fruit cultivars using principle component analysis (PCA) based on the phenolic profiles in the peach peel (**B**).

The phenolic profiles in the fruit peel showed variations between melting peaches and nectarines (Figure 1A,B). Compared with the 13 melting peach cultivars, the four nectarine cultivars contained relatively higher amounts of Q3GAL and Q3GLU (Figure 1A and Table 3). Furthermore, PCA also showed a clear distinction between the 13 melting peach cultivars (cluster A) and the four nectarine cultivars (cluster B) (Figure 1B). The first two PCs explained 48.9% and 27.9% of the variance, respectively. Interestingly, cluster A could be further divided into two subgroups, *i.e.*, clusters A1 and A2. Cluster A1 included all seven melting peach cultivars grown in the Zhejiang province, which were relatively higher in B1 content, while cluster A2 included all six melting peach cultivars grown in Shanghai, which exhibited relatively higher contents of Q3R and K3R (Figure 1B).

### 2.5. Total Phenolics and Antioxidant Capacities

The total phenolic contents in the peel and pulp extract of 17 peach cultivars were measured, and the antioxidant capacities of these fruits were evaluated using 2,2-diphenyl-1-picrylhydrazyl (DPPH) and ferric reducing antioxidant power (FRAP) methods. Obvious variations in the total phenolic contents, ranging from 4.58 to 12.68 mg gallic acid equivalent (GAE)/g DW in the peel and from 0.82 to 6.52 mg GAE/g DW in the pulp, were observed among the cultivars tested (Table 5). This variation was consistent with the previous results of Cheng and Crisosto [25] and Manzoor *et al.* [16]. In the peel, HY002 showed the highest total phenolic content, followed by HY018 and XY, while ANSM showed the lowest total phenolic content. In the pulp, XY showed the highest total phenolic content, followed by YL, while ZX showed the lowest total phenolic content.

The DPPH assay has been widely used to evaluate the free radical scavenging constituents in plants. The DPPH values for the different cultivars analysed varied from 6.35 to 19.84 mg trolox equivalent antioxidant capacity (TEAC)/g DW in the peel and from 1.05 to 15.01 mg TEAC/g DW in the pulp (Table 5). XY showed the highest DPPH values, while ZX showed the lowest DPPH values in both the peel and pulp tissues. In the peel, DPPH values in all nectarines tested were higher than the average value (14.6 mg TEAC/g DW) obtained for the 17 peach cultivars, consistent with the higher phenolic content in these fruits. In addition, much higher levels of DPPH radical scavenging activity were observed in the peel fraction compared with the pulp fraction, consistent with the previous results of Manzoor *et al.* [16].

The FRAP values of the peach cultivars varied from 3.24 to 13.85 mg TEAC/g DW in the peel and from 0.61 to 6.99 mg TEAC/g DW in the pulp. Both the peel and pulp of XY showed the highest FRAP values among all samples tested (Table 5). Similarly, the FRAP values in nectarine peels were higher than the average FRAP values (9.1 mg TEAC/g DW) obtained for the 17 peach cultivars. Higher FRAP values were observed for peel extracts compared with pulp extracts, consistent with the results of Guo *et al.* [26].

Since the two methods used above showed different antioxidant capacities for the same cultivar (Table 5), an overall antioxidant potency composite (APC) index was calculated for each cultivar according to the method of Seeram *et al.* [27]. The APC index showed obvious variations, ranging from 28.29 to 99.57 in the peel and 7.92 to 99.89 in the pulp (Table 5). XY, a melting peach cultivar, showed the highest APC index in both peel and pulp tissues and is thus an ideal peach cultivar for future breeding.

**Table 5:** Total phenolic contents and antioxidant activities in the peel and pulp extracts of 17 *P. persica* cultivars.

Cultivars	Peel					Pulp				
	Total Phenolics mg GAE/g DW	DPPH mg TEAC/g DW	FRAP mg TEAC/g DW	APC	Rank	Total Phenolics mg GAE/g DW	DPPH mg TEAC/g DW	FRAP mg TEAC/g DW	APC	Rank
ANSM	4.58 ± 0.06 ^j^	6.66 ± 0.27 ^h^	3.24 ± 0.01 ^j^	28.36	16	2.82 ± 0.13 ^i^	4.42 ± 0.06 ^e^	1.96 ± 0.08 ^h^	28.92	14
CF	9.54 ± 0.11 ^g^	13.83 ± 0.34 ^d^	7.70 ± 0.21 ^g^	62.34	12	4.09 ± 0.12 ^d,e^	6.56 ± 0.24 ^c^^,d^	3.26 ± 0.01 ^e^	45.47	7
CY	11.19 ± 0.12 ^d,e^	17.15 ± 1.01 ^b^	12.41 ± 0.13 ^b^	87.72	4	5.89 ± 0.02 ^c^	9.75 ± 0.62 ^b^	6.89 ± 0.04 ^a^	82.02	2
DX	10.12 ± 0.12 ^f^	15.53 ± 0.10 ^c^	11.17 ± 0.13 ^d^	79.16	8	4.19 ± 0.08 ^d^	7.17 ± 0.18 ^c^	4.95 ± 0.13 ^c^	59.63	4
DYBF	9.60 ± 0.02 ^g^	15.13 ± 0.26 ^c^	10.98 ± 0.07 ^d^	77.47	9	3.65 ± 0.05 ^f^	5.96 ± 0.11 ^d^	4.06 ± 0.07 ^d^	49.11	6
HJML	9.72 ± 0.34 ^f^^,g^	15.03 ± 0.95 ^c^	8.69 ± 0.21 ^f^	69.01	11	3.33 ± 0.08 ^g^	6.28 ± 0.10 ^c^^,d^	3.09 ± 0.05 ^e^	43.34	8
JHDBT	6.20 ± 0.17 ^h^	9.45 ± 0.71 ^f^	4.70 ± 0.04 ^h^	40.63	14	2.35 ± 0.07 ^k^	4.28 ± 0.41 ^e^	1.97 ± 0.05 ^h^	28.67	15
QSBT	11.06 ± 0.2 ^1,e^	19.79 ± 0.88 ^a^	11.71 ± 0.25 ^c^	91.73	3	3.59 ± 0.29 ^f^	6.46 ± 0.70 ^c^^,d^	3.89 ± 0.29 ^d^	49.75	5
SZZS	6.32 ± 0.23 ^h^	8.20 ± 0.47 ^g^	4.19 ± 0.10 ^i^	35.64	15	2.13 ± 0.01 ^l^	3.00 ± 0.12 ^f^	1.45 ± 0.02 ^i^	20.55	16
WJZBF	9.82 ± 0.23 ^f^^,g^	12.79 ± 0.11 ^e^	7.59 ± 0.18 ^g^	59.41	13	3.93 ± 0.07 ^e^	5.73 ± 0.19 ^d^	3.23 ± 0.03 ^e^	42.43	9
XY	12.25 ± 0.76 ^a^^,b^	19.84 ± 0.48 ^a^	13.85 ± 0.66 ^a^	99.57	1	6.52 ± 0.15 ^a^	15.01 ± 2.36 ^a^	6.99 ± 0.18 ^a^	99.89	1
YL	10.78 ± 0.43 ^e^	16.98 ± 0.12 ^b^	8.78 ± 0.69 ^f^	74.22	10	6.08 ± 0.10 ^b^	9.58 ± 0.22 ^b^	5.17 ± 0.16 ^b^	69.22	3
ZX	5.15 ± 0.04 ^i^	6.35 ± 0.63 ^h^	3.44 ± 0.13 ^j^	28.29	17	0.82 ± 0.03 ^m^	1.05 ± 0.07 ^g^	0.61 ± 0.01 ^j^	7.92	17
HY002	12.68 ± 0.14 ^a^	17.63 ± 0.14 ^b^	11.72 ± 0.15 ^c^	86.41	5	3.03 ± 0.07 ^h^	4.42 ± 0.15 ^e^	2.69 ± 0.04 ^f^	34.19	11
HY003	11.54 ± 0.06 ^c^^,d^	17.43 ± 0.15 ^b^	10.49 ± 0.07 ^e^	81.47	7	2.87 ± 0.03 ^h,i^	4.26 ± 0.35 ^e^	2.61 ± 0.03 ^f^	32.94	12
HY004	11.94 ± 0.04 ^b,c^	16.81 ± 0.50 ^b^	11.25 ± 0.32 ^c^^,d^	82.64	6	2.59 ± 0.02 ^j^	3.59 ± 0.14 ^e,f^	2.41 ± 0.04 ^g^	29.41	13
HY018	12.61 ± 0.12 ^a^	19.29 ± 0.58 ^a^	12.49 ± 0.09 ^b^	93.32	2	3.35 ± 0.02 ^g^	5.59 ± 0.12 ^d^	3.13 ± 0.03 ^e^	41.29	10

Data were expressed as the means ± standard deviation of triplicate samples; Different superscripts in the same column represent significant differences (*p* < 0.05); Antioxidant index score = ((sample score/best score) × 100).

### 2.6. Correlations between Fruit Bioactivity Traits

Correlation analyses were performed to investigate the relationship between the antioxidant capacity and the phenolic content in different peach samples (Table 6).

**Table 6:** Pearson’s correlation coefficients between antioxidant activities and phenolic contents.

Antioxidant Capacities/Phenolic Contents	Peel		Pulp	
	DPPH	FRAP	DPPH	FRAP
FRAP	0.952 **	1	0.918 **	1
Total phenolics	0.956 **	0.936 **	0.935 **	0.950 **
TIP	0.863 **	0.876 **	0.829 **	0.953 **
Hydroxycinnamates	0.881 **	0.919 **	0.642 **	0.802 **
Flavan-3-ols	0.942 **	0.938 **	0.827 **	0.907 **
Anthocyanin	0.473 **	0.408 **	0.062	0.071
Flavonols	0.338 *	0.329 *	−0.097	−0.124
NCHA	0.788 **	0.851 **	0.698 **	0.687 **
CHA	0.874 **	0.905 **	0.519 **	0.752 **
B1	0.675 **	0.676 **	0.873 **	0.925 **
CAT	0.907 **	0.891 **	0.550 **	0.718 **

Abbreviations: TIP, total individual phenolic; NCHA, neochlorogenic acid; CHA, chlorogenic acid; B1, procyanidin B1; CAT, Catechin; One and two asterisks represent statistical significance at *p* < 0.05 and *p* < 0.01, respectively.

High correlations between DPPH and FRAP were observed for both the peel (*r* = 0.952, *p* < 0.01) and the pulp (*r* = 0.918, *p* < 0.01), thereby validating these two methods for evaluating antioxidant activity. In addition, in both fruit tissues, the total phenolic content was strongly correlated with antioxidant activity, *i.e.*, extracts with higher total phenolic contents showed higher antioxidant activity, and *vice versa*. These data were consistent with the results of previous studies [11,16]. In addition, total individual phenolics (TIP, *i.e.*, the sum of nine individual phenolic compounds identified in this study), hydroxycinnamates, and flavan-3-ols also showed a significant correlation with the antioxidant activities in both tissues of the samples analysed (ranged from 0.642 to 0.942, *p* < 0.01). Flavonols and anthocyanins, however, did not show a good correlation with antioxidant activities. Among the nine phenolic compounds, NCHA, CHA, B1, and CAT showed a good correlation with the antioxidant activities of the fruit extracts (Table 6), which were mainly due to their relatively high concentrations in the fruit and their high intrinsic antioxidant activities (data not shown). Other compounds, such as C3G, were present at low concentrations in peach fruits and did not show a high correlation with the antioxidant activities in the fruit extracts.

## 3. Experimental Section

### 3.1. Chemicals

NCHA, CHA, B1, CAT, C3G, Q3GAL, Q3GLU, Q3R, K3R, GA standards, DPPH•, Trolox, 2,4,6-tris(2-pyridyl)-s-triazine (TPTZ), Folin-Ciocalteu reagent (2 mol/L), and acetonitrile were purchased from Sigma-Aldrich (St. Louis, MO, USA), and all chemicals were of chromatography grade. Double-distilled water (ddH_2_O) was used in all experiments, and samples for HPLC were filtered through a 0.22 μm membrane prior to injection. All other reagents were of analytical grade (Sinopharm Chemical Reagent Co., Ltd., Shanghai, China).

### 3.2. Materials

Thirteen melting peach and four nectarine cultivars were harvested at optimum maturity based on uniformity of shape and colour, absence of disease and mechanical damage from two Germplasm Collections in southern China during the summer of 2013 (Table 1). Specifically, melting peach cultivars ANSM, CF, JHDBT, SZZS, WJZBF, and ZX and nectarine cultivars HY002, HY003, HY004, and HY018 were harvested from Zhuanghang Integrated Experiment Station, Shanghai Academy of Agricultural Sciences, Shanghai, China (Latitude 30°53'31.79''N, Longitude 121°23'6.45''E). The remaining melting peach cultivars, CY, DX, DYBF, HJML, QSBT, XY, and YL, were harvested from Fenghua Honey Peach Institute, Ningbo, Zhejiang, China (Latitude 29°39'30.92''N, Longitude 121°24'25.18''E). After harvest, the fruits were separated into two groups, *i.e.*, peel and pulp, and frozen in liquid nitrogen. After freeze-drying (FM 25EL-85, VirTis, Gardiner, NY, USA), all samples were ground into a fine powder and stored at −80 °C until extraction and analysis of phenolics.

### 3.3. Fruit Quality Analysis

Twelve fruit of each cultivar were randomly selected, and quality traits, such as FW, FSI, SSC, were measured. The flesh colour was recorded as white, yellow, and red. The height and diameter at the widest point of the fruit were measured using a vernier calliper, and the height/diameter ratio was calculated for FSI. SSC was measured using a digital refractometer (Atago PR-101R, Tokyo, Japan), and the data were expressed as °Brix.

### 3.4. Preparation of Fruit Peels and Pulp Extracts

The ground fruit powder (0.30 g) was extracted in 3 mL of 80% methanol through sonication (DKZ-2B, Shanghai, China) for 30 min. The ultrasonic frequency and power were 60 kHz and 30 W, respectively. The extracts were centrifuged at 8000 rpm for 10 min at 4 °C and the residue was extracted twice as described above. Both supernatants were combined and used for the determination of phenolic compounds and antioxidant activity.

### 3.5. Determination of Total Phenolics

Total phenolics in the fruit extracts were measured using a modified colorimetric Folin-Ciocalteu method [28]. Four millilitres of ddH_2_O and appropriately 0.5 mL of diluted fruit extracts were placed in a test tube. Folin-Ciocalteu reagent (0.5 mol/L, 0.5 mL) was added to the solution, and the reaction was incubated for 3 min. The reaction was neutralized with 1 mL of saturated sodium carbonate. After 2 h, the absorbance at 760 nm was measured using a spectrophotometer (UV-2550, Shimadzu, Tokyo, Japan). GA was used as a standard, and the data were expressed as mg GAE/g DW.

### 3.6. HPLC-DAD and LC-ESI-MS/MS Analysis of Phenolic Compounds

Individual phenolic compounds were firstly analysed through HPLC (2695 pump, 2996 diode array detector, Waters) coupled with an octadecyl silane (ODS) C18 analytical column (4.6 × 250 mm). The flow rate was 1 mL/min, with a column temperature of 25 °C and an injection volume of 10 μL. The compounds were detected between 200 and 550 nm. The mobile phase of HPLC comprised 0.1% (*v*/*v*) formic acid in water (eluent A) and 0.1% formic acid in acetonitrile (eluent B), according to Scordino *et al.* [17], with some modifications. The following gradient programme was used: 0 min, 5% B; 50 min, 28% B; 60 min, 43% B; 60–65 min, 43% B; 70–75 min, 5% B.

Mass spectrometric analyses were performed by an Agilent 6460 triple quadrupole mass spectrometer equipped with an ESI source (Agilent Technologies, Santa Clara, CA, USA) that operated in both positive and negative ionization mode. The nebulizer pressure was set to 45 psi and the flow rate of drying gas was 5 L/min. The collision energy was set to 5, 15, 25 and 35 eV. The flow rate and the temperature of the sheath gas were 11 L/min and 350 °C, respectively. Chromatographic separations were done on an ODS C18 analytical column (4.6 × 250 mm) using an Agilent 1290 Infinity HPLC system (Agilent Technologies, Santa Clara, CA, USA). The eluent was split and approximately 0.3 mL/min was introduced into the mass detector. The data acquisition and processing were carried out on an Agilent Mass Hunter Workstation.

Individual phenolic compounds were quantified using the respective standard curves: NCHA and CHA were detected at 325 nm; B1 and CAT were detected at 280 nm; C3G was detected at 517 nm; and Q3GAL, Q3GLU, Q3R, and K3R were detected at 350 nm, and data were expressed as mg/kg DW.

### 3.7. Antioxidant Activity Assays

DPPH radical scavenging activity was measured according to Brand-Williams *et al.* [29], with modifications. The reaction, containing 2 μL of sample and 198 μL of 25 μg/mL DPPH solution, was incubated at room temperature for 60 min. Subsequently, the absorbance of samples was measured at 517 nm using a microplate reader (Synergy H1, Biotek, Winooski, VT, USA). Trolox was used as a standard, and the data were expressed as mg TEAC/g DW.

The FRAP was measured according to Benzie and Strain [30], with modifications. A fresh working solution, containing 100 mL of 300 mmol/L acetate buffer (pH 3.6), 10 mL of 10 mmol/L TPTZ solution in 40 mmol/L of HCl, and 10 mL of 20 mmol/L FeCl_3_ solution, was prepared, and 0.1 mL of sample was added to 0.9 mL of the FRAP solution, followed by incubation for 10 min at 37 °C. Subsequently, the absorbance at 593 nm was measured using a spectrophotometer. Trolox was used as a standard, and the data were expressed as mg TEAC/g DW.

### 3.8. Statistical Analysis

In addition to the fruit quality index in Table 1, measured in 12 fruits for each cultivar, all other data were obtained from at least three replications and were expressed as the means ± standard deviation. The statistical analysis was performed using SPSS 17.0 software (SPSS Inc., Chicago, IL, USA), and significant differences among the samples were calculated using one-way ANOVA, followed by Duncan’s multiple range test at *p* < 0.05. Pearson correlation coefficients were calculated between antioxidant activity and phenolic contents at *p* < 0.05. PCA was performed using the MetaboAnalyst platform [31].

## 4. Conclusions

In the present study, the phenolic contents and antioxidant activities in the peel and pulp of 13 melting peach cultivars and four nectarine cultivars grown in southern China were investigated. Nine phenolic compounds were identified and quantified using their authentic standards. In general, CHA and CAT were the predominant components detected in both tissues. The peel contained higher amounts of phenolics than the pulp, and anthocyanins and flavonols were primarily detected in the peel. Based on phenolic profile in the peel, our results showed a clear distinction between phenolics in different peach types, which indicated the potential application of phenolic compounds in peach classifications as well as breeding. In addition, the APC index of different cultivars varied from 28.29 to 99.57 in the peel and from 7.92 to 99.89 in the pulp, and the highest value in both the peel and pulp was observed in the XY melting peach cultivar. Correlation analyses showed that peach cultivars rich in hydroxycinnamates (NCHA and CHA) and flavan-3-ols (B1 and CAT) showed relatively higher antioxidant activities. Our findings provide useful information for future study and utilization of the peach germplasm in China.
